# Variable mating behaviors and the maintenance of tropical biodiversity

**DOI:** 10.3389/fgene.2015.00183

**Published:** 2015-05-19

**Authors:** Charles H. Cannon, Manuel Lerdau

**Affiliations:** ^1^Key Lab in Tropical Ecology, Xishuangbanna Tropical Botanical Garden, Chinese Academy of Sciences, Menglun, China; ^2^Department of Biological Sciences, Texas Tech University, Lubbock, TX, USA; ^3^Departments of Environmental Sciences and Biology, University of Virginia, Charlottesville, VA, USA

**Keywords:** syngameon, genomic mutualists, tropical trees, selfing, inter-specific hybridization, density-dependence, maintenance of diversity

## Abstract

Current theoretical studies on mechanisms promoting species co-existence in diverse communities assume that species are fixed in their mating behavior. Each species is a discrete evolutionary unit, even though most empirical evidence indicates that inter-specific gene flow occurs in plant and animal groups. Here, in a data-driven meta-community model of species co-existence, we allow mating behavior to respond to local species composition and abundance. While individuals primarily out-cross, species maintain a diminished capacity for selfing and hybridization. Mate choice is treated as a variable behavior, which responds to intrinsic traits determining mate choice and the density and availability of sympatric inter-fertile individuals. When mate choice is strongly limited, even low survivorship of selfed offspring can prevent extinction of rare species. With increasing mate choice, low hybridization success rates maintain community level diversity for extended periods of time. In high diversity tropical tree communities, competition among sympatric congeneric species is negligible, because direct spatial proximity with close relatives is infrequent. Therefore, the genomic donorship presents little cost. By incorporating variable mating behavior into evolutionary models of diversification, we also discuss how participation in a syngameon may be selectively advantageous. We view this behavior as a genomic mutualism, where maintenance of genomic structure and diminished inter-fertility, allows each species in the syngameon to benefit from a greater effective population size during episodes of selective disadvantage. Rare species would play a particularly important role in these syngameons as they are more likely to produce heterospecific crosses and transgressive phenotypes. We propose that inter-specific gene flow can play a critical role by allowing genomic mutualists to avoid extinction and gain local adaptations.

## Introduction

Numerous ecological mechanisms, both stochastic and deterministic, affect species co-existence in communities with high levels of diversity (Connell, 1961; Janzen, 1970; Hubbell, 1997; Webb and Peart, 1999; Wright, 2002; Volkov et al., 2005). While most attention has focused on how such diversity arises and how interactions among species promote and constrain diversity, fewer studies have examined the implications of demographic and genetic factors in the maintenance of species-level diversity. The unified neutral theory of biodiversity and biogeography (Hubbell, 2001; Rosindell et al., 2011) has provided an effective set of ecological null models to test patterns of species composition and abundance in these communities and to discern the roles of different ecological mechanisms in promoting/constraining species-diversity.

In these ecological models, species act as discrete evolutionary units: conspecific individuals are inter-changeable while heterospecific individuals are completely distinct. Speciation, in other words, is assumed to be complete and reproductive isolation fixed. While only two studies have investigated tropical trees, some studies on diverse groups of animals and plants have shown, however, that closely related and sympatric species often maintain some level of inter-specific fertility, particularly among plants (Anderson, 1953; Morjan and Rieseberg, 2004; Seehausen, 2004; Arnold et al., 2008b; Givnish, 2010). Numerous studies of secondary contact zones have demonstrated that hybrid offspring can invade new habitats (Donovan et al., 2010), inter-specific gene flow can allow the introgression of advantageous alleles from one species to another (Arnold et al., 2008a,b; Fitzpatrick et al., 2010), and that genetic rescue through hybridization can occur during population crashes (Baskett and Gomulkiewicz, 2011). Increasing genome-scale evidence has also demonstrated that reticulation among sympatric close-relatives has played a major role in the few well-studied groups (Dasmahapatra et al., 2012; Twyford and Ennos, 2012; Yoder et al., 2013; Thomson et al., 2015). Self-fertilization, primarily in plants, can also play a significant role in reproduction (Porcher and Lande, 2005).

In this study, using a numerical simulation model, we attempt to incorporate an ecologically responsive mating behavior by allowing both inter-specific crosses and self-fertilization to play a role in neutral meta-community ecological dynamics. We combine both intrinsic levels of inter-fertility and self-compatibility with extrinsic community-level density-dependent factors, e.g., species composition and abundance. While ecologists have achieved a relatively good understanding of ecological mechanisms for species co-existence, we have little understanding of how species diversify in tropical communities. This analysis allows us to bring traditional perspectives in plant genetics and evolution, like the syngameon (Lotsy, 1925; Grant, 1971), to bear on the traditionally ecological issue of biodiversity in hyperdiverse communities.

## Variable Mating Behaviors

We model individual plant reproductive behaviors, namely selfing, conspecific out-crossing, and inter-specific gene flow, in a meta-community of varying species diversity. We allow these behaviors to respond to an environmental signal, which is the composition and abundance of pollen reaching their stigma. as responsive and adaptive reproductive behaviors. These three reproductive behaviors are common in wild populations and individuals commonly retain the capacity for each behavior. Although most tropical trees are highly out-crossed (Bawa et al., 1985a,b), selfing may be more frequent than expected (Kondo et al., 2011), particularly for endemic species (Alonso et al., 2010). Selfing rates have been shown to be inversely related to population densities (Ward et al., 2005). Moreover, sympatric and closely-related species can and do hybridize in natural communities (Kamiya et al., 2011; Duminil et al., 2012; Thomson et al., 2015).

Importantly, genome size appears to be largely stable in tropical trees (Chen et al., 2014). Polyploidization is generally considered rare among woody plants (Petit and Hampe, 2006). Most genera that have been examined have consistent ploidy levels while the impact of homoploid hybridization on diversification in plants has been reviewed (Rieseberg and Carney, 1998). Recent studies indicate that the genomes of eucalypt species are largely co-linear (Hudson et al., 2012). Conserved genomic structure facilitates variable mating behaviors while unstable genomic structure should promote rapid speciation. In the following discussion, we refer to heterospecifics that retain partial inter-fertility as “genomic mutualists.” This idea corresponds to the genic model of speciation (Wu, 2001), corresponding to a stable and intermediate inter-fertility (stages II or III) where speciation never reaches completion. This model is based upon the basic structure proposed for a syngameon (Lotsy, 1925; Grant, 1971), where multiple species remain partially inter-fertile but primarily experience divergent selection on portions of the genome with low levels of neutral or adaptive gene flow occuring in other parts of the genome.

Highly diverse communities would therefore be composed of numerous syngameons or suites of genomic mutualists, where individuals predominantly out-cross but retain some diminished capacity for self-fertilization or inter-specific hybridization against a conserved genomic background. Variable mating behavior in these individuals, instead of being determined solely by genotype, is determined by the quantity and quality of pollen rain. The quantity of a particular species of pollen, either from conspecifics or genomic mutualists, could act as a proxy measure for “success” in the community of that particular genotype, either because of its capture of pollination services or its eco-physiological success in that particular location. Pollen precedence would control this behavior, where conspecific pollen is more vigorous (Williams et al., 1999) and, if present, will fertilize most ovules (Howard et al., 1998). On the other hand, if an individual primarily receives heterospecific pollen, despite reduced vigor and lower fertilization rates, the probability of hybridization correspondingly increases. This type of density-dependent, unidirectional gene flow between sympatric genomic mutualists has been reported in oaks (Lepais et al., 2009). While some plants exhibit some level of self-incompatibility, the selective advantage of self-fertilization typically remains high, despite potential costs of inbreeding depression (Steinbachs and Holsinger, 1999). In situations where conspecific pollen is rare, possible mentor pollen effects could also facilitate hybridization (Knox et al., 1972), as mixtures of self and hetero-specific pollen on the stigma have been found to make the pistil more receptive to hybridization.

Our model incorporates an ecologically dynamic framework of conditions that determine species behavior based on the interaction of several probabilistic realities. This variable behavior is akin to fuzzy reasoning (Zadeh, 2008), also known as conditional logic, which remains somewhat controversial although it has also been successfully applied in the ecological modeling of animal behavior (Inglis and Langton, 2006). These principles are particularly advantageous when uncertainty and contingent processes play a significant role in the outcome. Given the general complexity of highly diverse tropical tree communities, particularly given their slow macro-evolutionary processes (Petit and Hampe, 2006) and dynamic biogeographic histories (Cannon et al., 2009), uncertainty should be expected to play a central role in the evolutionary and ecological behavior of tropical organisms. In these evolutionary and ecological conditions, variable mating behavior, where mate choice is contingent upon the environmental signal in the pollen rain, should be an appropriate model.

## Resisting the Extinction Vortex

Because a large proportion of species in highly diverse communities are rare (He et al., 1997), most are chronically vulnerable to stochastic local extinction (Ovaskainen and Meerson, 2010). This vulnerability implies either that extinction (and the concomitant immigration and/or speciation) rates are high in such systems or that rare species have coping mechanisms to avoid local extinction. Variable mating behaviors would be a potential coping mechanism because isolated individuals, suffering from an absence of conspecifics and poor pollination service in the local neighborhood, could expand their effective population sizes. Unidirectional gene flow into the rare species could be disadvantageous to individuals of the dominant species if the two species were in direct ecological competition. In highly diverse communities, however, most species form only a small fraction of the entire community. This general feature of highly diverse communities could ameliorate the potential cost of being a genetic donor to a rare species, because direct competition with hybrid offspring clustered around the rare genetic recipient would be correspondingly rare. Previous analysis of density dependent competition at the seedling stage in lowland tropical forest on Barro Colorado Island demonstrated that the individuals were affected by conspecific but not heterospecific competitors (Comita et al., 2010), but because the majority of species are sympatric with closely-related species, these results could underestimate the potential competition among genomic mutualists.

**FIGURE 1:**
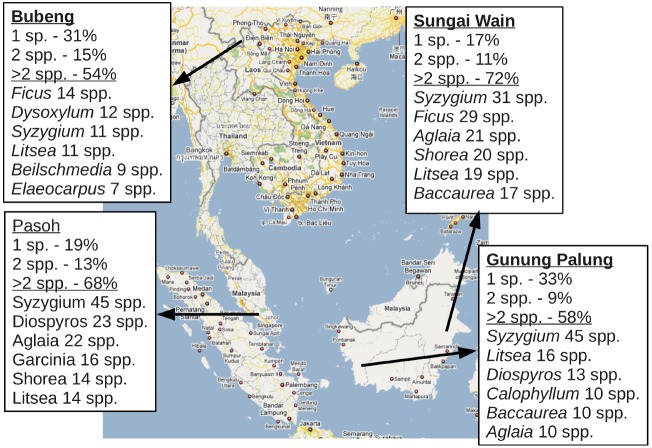
**Percentage of species existing in sympatry with congenerics and species richness of most diverse tree genera in four locations across tropical Asia.** “1 sp.” illustrates the number of species with a only one species from a genus present in the community; “2 spp.” illustrates the number with species with at least two sympatric congeneric; and “>2 spp.” illustrates the number of species with at least three sympatric congenerics. The five most diverse genera in each location are listed below the solid line, with the total number of species observed indicated.

To explore the potential costs to common species of unidirectional hybridization with rare species through direct competition with their hybrid offspring, we examine conspecific and congeneric encounter rates within local neighborhoods in the Pasoh Long-Term Dynamics plot (Kochummen and LaFrankie, 1990), a lowland rainforest in peninsular Malaysia. At Pasoh, only 157 out of 811 species (19%) were the sole representative of their genus in the local community, while another 106 species were sympatric with one other congeneric species. The remaining 548 species (68%) exist sympatrically with at least two other congeners. This pattern is evident in numerous other locations across Southeast Asia (Figure 1). The opportunity for variable mating behavior is therefore abundant in these communities.

Here, we incorporate variable mating behavior into a meta-community model of species co-existence using a data-based simulation with a simple decision tree based on the quantity and species composition of pollen received by an individual. We assume that among genomic mutualists, pollination is a stochastic process and the composition of the pollen rain is determined by the relative population densities of inter-fertile individuals in the meta-community. Additionally, we assume that conspecific out-crossing is substantially more likely to produce viable offspring than selfing or hybridization but if a cross is successful, the offspring are ecologically equivalent. Here, the assumptions of ecological equivalence among a suite of closely-related species is probably more acceptable than the assumption of community wide ecological equivalence across all of the species in the community made in the neutral theory of biodiversity and biogeography (Hubbell, 2001). Even if hybrids and selfed offspring have lowered fitness, the alternative is extinction, which is always more costly. We believe that modeling reproductive effort as a variable behavior, controlled by the density-dependent local community composition of inter-fertile species and the probability of successful outcomes from various types of crosses, can provide further insight into species extinction in highly diverse communities, generate numerous testable hypotheses about the evolution of rare species, and has major implications for species management.

## Materials and Methods

### Model Parameters

We included six variable parameters in our model of variable mating behavior and species co-existence. We performed three replicates of all possible combinations of the variable parameters. The variable parameters were:

#### Species Diversity

The initial diversity of genomic mutualists that occur within the larger matrix of the community. We assume that all species are in the same genus and experience the same level of self-compatibility and inter-fertility. The values included 3, 5, and 10. These values capture the majority of the observed range of local diversity of congeneric taxa in the Pasoh 50 ha plot (see below).

#### Community Size

The numbers of individuals in the community of genomic mutualist at the three diversity levels. The relative abundances of each species are based on the patterns observed in Pasoh forest plot data for groups of congeners. 3 spp. = {195,75,30}; 5 spp. = {300, 200, 150, 100, 50}; 10 spp. = {220, 200, 125, 110, 100, 90, 75, 50, 20, 10}.

#### Pollen Limitation Coefficient

Proportion of the community of genomic mutualist that act as pollen donors in any one reproductive event. For example, in the three species community simulation where the total population is 300, with a PLC = 0.1, a single individual receives pollen from thirty other individuals (0.1 × 300); in the five species community simulation where the total population is 800, with a PLC = 0.01, a single individual receives pollen from eight other individuals (0.01 × 800). This parameter had a large impact on reproductive success and was examined across coefficient values ranging from 0.01 to 0.5 {0.01, 0.015, 0.02, 0.025, 0.1, 0.15, 0.25, 0.5}.

#### Self-fertilization Success

The probability of producing a viable offspring from a self-fertilization event. Conspecific crosses were always successful. A value of 0 signifies complete self incompatibility. These values are derived from empirical studies across tropical tree taxa. {0, 0.1, 0.25, 0.4}.

#### Hybridization Success

The probability of producing a viable offspring from an inter-specific pollination event. A value of 0 signifies complete hybrid incompatibility. These values of interfertility among closely-related species are derived from previously cited reviews of plant species {0, 0.1, 0.25, 0.4}.

#### Individual Fecundity

The number of potential progeny that could be produced by an individual in each reproductive event {10, 50, 250}, less the unsuccessful self-fertilization and inter-specific crosses. It should be noted that this level of fecundity greatly underestimates the actual fecundity of almost any tropical tree species, which can easily produce orders of magnitude greater ovules. This underestimate of fecundity simplified and facilitated rapid computation of the simulation. This very limited potential fecundity should impose a very strong burden on the fraction of successful hybrids or selfed offspring.

Several parameters were not allowed to vary in our simulations. We assumed that all crosses between conspecific individuals were successful and that all viable progeny were equally fit and ecological equivalent, whether they were of self, hybrid, or out-crossed origin. All individuals were bisexual and species identity of the progeny was determined by the mother. The size of the modeled community was fixed through the simulation (see parameter 2). Stochastic mortality was fixed at 1.5%. Note that individual reproductive success and the type of offspring produced can only be determined by the interaction of several probabilistic parameters and community composition and not inherent to the individual. No spatial parameters were imposed on the model, therefore any individual could mate with any other individual in the community with equal probability.

### Simulation Procedure

We established the initial community for each of three replicates for all unique combinations of variable parameters (see above). At the outset of the model, a community of a fixed population size was established (3 spp. = 300 individuals; 5 spp. = 800 inds., and 10 spp. = 1000 inds), composed of “common” and “rare” species (see parameter 2). For every individual in the initial community, three values were recorded and tracked through each reproductive event for the entire simulation. These values were: (1) individual identity, (2) species identity, and (3) age.

Each simulation was conducted in an iterative process of three steps (see below). Reproduction was simultaneous for all individuals over 500 events and no spatial effects were incorporated into the model. During each reproductive event, each individual was crossed with a random selection of individuals in the community, irrespective of species, given the values of the pollination limitation coefficient and fecundity parameters. Recruitment was random from the entire pool of viable progeny and equal to the number of stochastic deaths. We did not attempt to model the entire community of nested sets of inter-fertile species but each small subset of sympatric and inter-fertile species was modeled separately. The mean number of species remaining in the community at the end of the simulation from the three replicates for all possible combinations of parameter values was used to generate a response surface to examine the sensitivity of the community at each parameter value across its range of variation.

### Step One

Generate the progeny for each individual, based upon the particular set of parameter values used in each run of the simulation. Progeny for each individual were generated during each reproductive event in the following way:

A random subset of individuals in the community were chosen as pollen donors, based upon the pollination limitation coefficient (parameter 3). No spatial parameters were imposed on mate selection.

1. All crosses between conspecifics produce a viable progeny.
2. A subset of inter-specific crosses produce viable progeny, based upon the probabilistic success of inter-specific crosses (parameter 5).
3. The total number of attempted crosses from #1 and #2 are subtracted from the individual fecundity (parameter 6), to obtain the number of remaining ovules for which pollination failed. Self-fertilization was then attempted for this remainder with the number of viable selfed offspring determined by the probabilistic success of self-fertilization (parameter 4).
4. The viable progeny produced by each individual in each reproductive event is therefore the sum of #1, 2, and 3, which will be some fraction of parameter 6.

### Step Two

A proportion of the existing individuals, chosen randomly, in the model die, given our rate of stochastic mortality (fixed at 1.5%). All viable progeny produced by all individuals in step one are pooled into a “seed bank.”

### Step Three

Individuals are chosen from the seed bank as recruits to replace the dead individuals. The seed bank was also substantially larger than the required number for replacement. These progeny are immediately able to reproduce. One “year” is added to each living individual’s age.

### Community Composition of Closely-related Species

To estimate the potential impact of inter-specific hybridization at the community level, we calculated the number of tree species that live sympatrically in the Pasoh Long-Term Dynamics Plot with congeneric species, assuming that congeneric species are at least partially inter-fertile and represent “genomic mutualists.” To estimate the potential cost of unidirectional gene flow to dominant species, we counted the number of conspecific and congeneric individuals present in the 25 m radius neighborhood surrounding 601 focal trees, representing 15 individuals from the 10 most common species from four categories of congeneric diversity: 3 species/genus, 3 species/genus, 5–6 species/genus, 10–14 species/genus, plus the most common species in the most diverse genus (45 *Eugenia* spp.). We tested empirical patterns against a null spatial model for local neighborhoods, using 1000 replicates. Simulations and analyses were written in Mathematica 7 (Wolfram Research, 2008).

## Results

**FIGURE 2:**
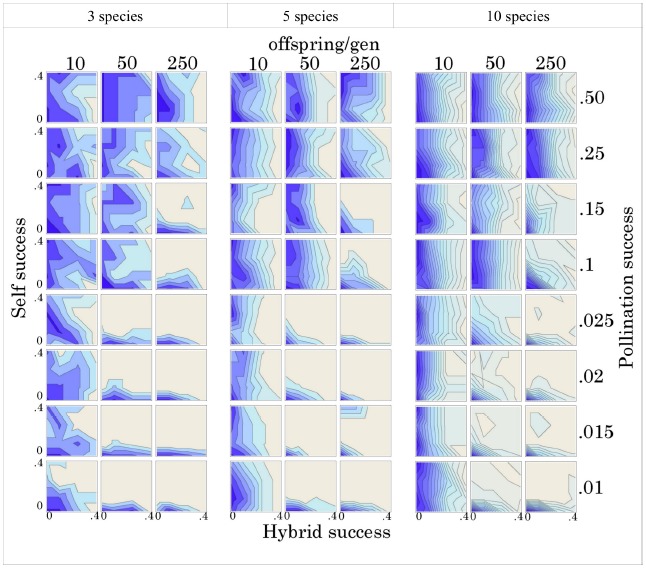
**Species richness after 500 reproductive events, given different levels of sympatry (3, 5, and 10 congeneric species), fecundity (10, 50, and 250 offspring/generation), pollination success (0.01, 0.015, 0.02, 0.025, 0.1, 0.15, 0.25, and 0.50), selfing success (0, 0.1, 0.25, and 0.4), and hybridization success (0, 0.1, 0.25, and 0.4).** Each contour line represents 0.5 species and darker shades indicate lower species diversity. Contour lines in each graph are interpolated from selfing and hybridization success rates. See Methods and Supplemental Information for more details.

Given stochastic mortality among individuals, we find that hybridization success, self-fertilization success, degree of pollination limitation, and level of fecundity all have significant effects on species co-existence (Figure 2). Inter-specific hybridization had the most pervasive effect, even at relatively low probabilities of success, maintaining the original community diversity over a substantial number (500x) of reproductive events, regardless of species diversity, pollination limitation, or fecundity. The effect becomes slightly stronger as diversity increases among the suite of genomic mutualists. The rate of hybridization success at which this effect becomes obvious (∼20%) is probably a substantial over-estimate, given the substantial under-estimate of individual fecundity in the model compared to the actual fecundity of trees. Tropical trees can produce massive amounts of ovules during each reproductive event. We would argue that the important factor is the absolute number of viable offspring produced and not the proportion. Given realistic levels of fecundity, we would suggest that vanishingly small levels of inter-fertility, probably below those measurable in a typical field study, would have a similar impact to those observed in this model.

When pollination limitation is strong (< = 10%) and fecundity is moderate to high (>10 offspring), given our model, self-fertilization strongly promotes species co-existence, even at low levels of success (10%). Again, the same statement could be made here about the importance of the absolute number of viable offspring and not the proportion. A bisexual individual only needs to produce a single offspring to be “fit.” Once pollination success improves, with more than 10% of the community acting as potential mates, the patterns become consistent and largely unresponsive to fecundity, selfing and hybridization success. On the other hand, when self-fertilization and hybridization are not allowed (x- and y- origin on all graphs), the communities in our model invariably lose all of their diversity through stochastic extinction of rare species and eventually consist of a single species.

**FIGURE 3:**
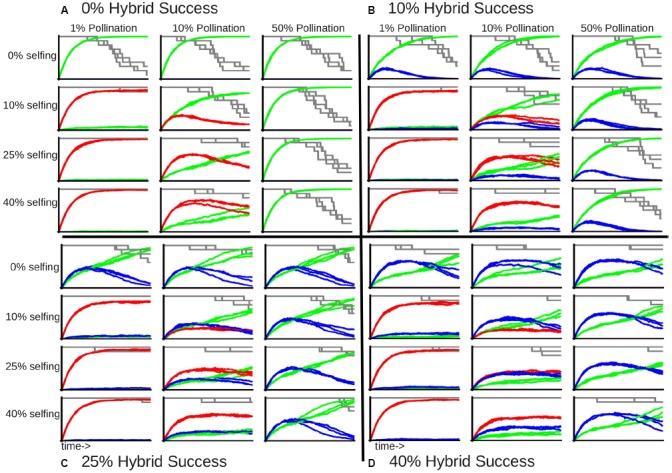
**Proportion of community recruitment derived from different types of mating, given an initial community containing 10 species with large reproductive capacity with varying levels of hybridization and selfing success.** Each large panel indicates the inter-specific hybridization success: **(A)** 0%; **(B)** 10%; **(C)** 25%; and **(D)** 40%. Each row in a single panel indicates the selfing success: 0, 10, 25, and 40%, top to bottom respectively. Each column in a single panel indicates the pollination success: 1, 10, and 50%, left to right respectively. The green line indicates the proportion of out-crossed offspring, the red line indicates selfed offspring, and the blue line indicates hybrid offspring. The gray line indicates fraction of remaining species richness, beginning with 10 species. Each graph represents three replicates for each set of mating parameters through 500 generations.

Self-fertilization became the dominant mode of reproduction when pollination limitation is strong (red lines in leftmost graphs in each panel of Figure 3), as almost all of the remaining individuals are a product of selfing at the end of the simulation, even when the success of selfed crosses is only 10%. On the other hand, when pollen limitation is not a factor and individuals can cross with half of the meta-population, selfed individuals never occur in the community (absence of red lines in rightmost graphs in each panel of Figure 3). The rate of inter-specific hybridization does not respond strongly to pollination limitation when selfing is not allowed (blue lines in the top row of graphs in each panel of Figure 3), although it does seem to interact with selfing, as increased pollinator success leads to greater proportions of inter-specific hybridization relative to selfing (Figure 3). Finally, the proportion of outcrossed individuals gradually increase with decreasing pollination limitation through the simulation, with the proportion of hybrid offspring reaching a peak and slowly tapering off (Figure 3). This pattern indicates that while variable mating behaviors delay extinction of rare species, they do not prevent extinction.

**FIGURE 4:**
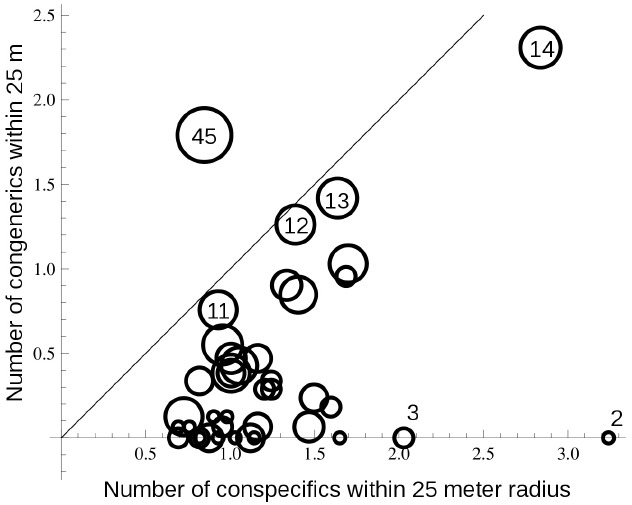
**The relationship between the number of conspecific and congeneric individuals in a local neighborhood with 25 m radius around focal trees.** Focal trees represent species with different numbers of sympatric congenerics in the Pasoh 50-ha plot. Each circle represents the mean values for 15 individuals from 61 of the most abundant species in each suite of sympatric congeners. The diameter of each circle indicates the number of congeneric species present in the community for each species. Several circles are labeled to indicate scale. The solid line illustrates a 1:1 ratio. Mean neighborhood size was 197 individuals composed of an average of 104 species.

In terms of possible costs to the common species, who would be acting as pollen donors, caused by direct competition with hybrid offspring, we found that direct competition among closely-related species rarely occurred within local neighborhoods in highly diverse forests simply because of the general low densities of species. In the Pasoh forest, the most common tree species accounts for less than 3% of the stems, while species with median levels of abundance contribute less than 0.04% to the entire community. Within a local neighborhood of 25 m radius, a focal individual encounters an average of 197 individuals, composed of an average of 104 species. The observed local neighborhood species diversity is significantly lower than the mean species diversity predicted by a null spatial model (134 spp., *p* < 0.05), agreeing with previous reports of clumped species distribution patterns (Condit et al., 2000). Overall, the number of stems >1 cm DBH of both conspecifics and congeners represents a very small fraction of the local neighborhood, and focal individuals are more likely to encounter conspecific individuals than congeners (Figure 4), although as congeneric diversity increases, the encounter rate becomes roughly equivalent for conspecifics and congenerics. Many individuals in genera with 2–3 sympatric species almost never encounter congeneric individuals. Given that individuals almost never directly compete with congenerics, unidirectional inter-specific hybridization, from dominant to rare species, cannot cause significant genetic costs to the pollen donor.

## Discussion

When variable mating behaviors are allowed in a stochastic meta-community model, species co-existence in diverse communities is greatly enhanced, even when fertility among species is low compared to out-crossing with conspecifics. In this neutral model, this result occurs primarily by delaying the local stochastic extinction of rare species. As their population size declines, the reproductive effort of rare species will be pushed toward hybridization and selfing because they no longer receive sufficient conspecific pollen. This shift in mating behavior not only effectively expands the local effective population size but it greatly increases their potential genetic diversification and allows them to capture adaptive genes present in locally dominant species. As the local population approaches extinction, variable mating behaviors become increasingly beneficial. Because of the limited direct interaction with congeneric individuals in the local neighborhood, the dominant species also incurs little cost to being a pollen donor to the rare species.

Seed dispersal limitation and clumped species distributions are a general characteristic of tropical tree communities (Ashton, 1969; Condit et al., 2000), which limits the recolonization of a community by rare species and creates spatially clumped “families”. First of all, these characteristics should further increase the likelihood of stochastic local extinction in rare species. Furthermore, the offspring of a rare species, including hybrids, will form a spatially clumped grove. The proximity of these mixed offspring could effectively allow them to back-cross, increasing mate choice with family members and potentially re-establish the local population (Baskett and Gomulkiewicz, 2011), returning the species to being predominantly out-crossed. It is possible that if the rare species did not recover locally, it would be swamped out by the common species. Given this model, rare species could also become a nexus of diversification as they begin to produce hybrid phenotypes, possibly transgressive or exadapted in a biotically and abiotically complex and dynamic environment (Givnish, 2010).

**FIGURE 5:**
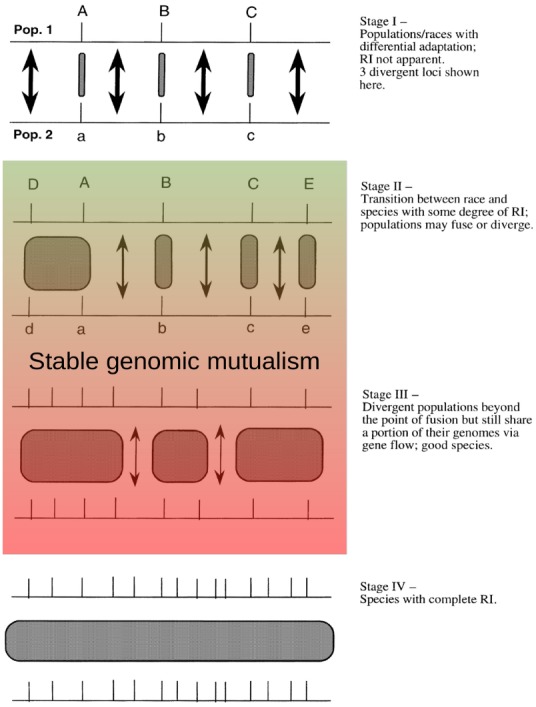
**Intermediate and diminished gene flow proposed among stable genomic mutualists as adapted from Wu’s (2001)**.

This behavior fits well with observations of inter-specific gene flow and the interaction of species in syngameons. We propose that a persistent but reduced capacity for variable mating behaviors among sympatric closely-related species would create a stable genomic mutualism (Figure 5). Adapted from Wu’s (2001) view of genic speciation, these genomic mutualisms represent a balance between purifying selection within a species for a particular phenotype and diversifying selection among species for novel phenotypes. Gene flow among these genomic mutualists would be relatively infrequent, highly variable in spatial scale, primarily unidirectional, and episodic, caused by either stochastic or deterministic population decline of one species in the local community. During these episodes, individuals of an increasingly rare species will primarily receive either hetero-specific or self pollen, greatly increasing the probability and advantage of producing offspring through these alternative reproductive pathways. As would be expected, selfing primarily benefits individuals when pollination limitation is extreme. Even if the average viability of selfed or hybrid offspring is low, a small proportion of individual offspring might have equal or even greater fitness than the mother tree, particularly if environmental factors are changing and creating novel habitats (Donovan et al., 2010). During these periods of local population decline, inter-specific hybridization can also provide selective advantages to rare species by allowing them to capture advantageous alleles and traits from successful species (Fitzpatrick et al., 2010). If a population is crashing due to deterministic processes, instead of stochastic reasons, such as susceptibility to fungal infection, then capturing genetic variation and alleles from a dominant and resistant genomic mutualist will be advantageous (Barton, 2001).

Diverse tree communities, exemplified by those in tropical Southeast Asia, exist in highly dynamic biogeographic, climatic, and ecological landscapes (Cannon et al., 2009; Woodruff, 2010; Slik et al., 2011; Raes et al., 2014), punctuated by brief periods of rapid change and strong selection that may dominate evolution (Gutschick and BassiriRad, 2003). High biological diversity itself lends a significant element of ecological complexity to the community, as the local composition of predators, herbivores, pollinators, and other inter-acting species at different trophic levels is variable spatially and temporally. Some studies indicate that known hybrid zones promote biodiversity in other parts of the community (Whitham et al., 1994; Adams et al., 2011). Ultimately, given the complexity of these communities, both in their current setting and in their historical dynamics, a high element of uncertainty exists in the reproductive success of an individual genotype, particularly given the general pollination limitation in these highly diverse systems (Alonso et al., 2010), which limits mate choice. In this initial formulation, our model focuses on tropical tree communities. Many of the properties of the model may also apply to other organisms living in species rich communities such as coral reefs, where most species are sympatric with closely related and partially inter-fertile species.

## Chronically Rare

In highly diverse communities, local rarity of the majority of species is a pervasive feature (Connell, 1978; Kochummen and LaFrankie, 1990; Pitman et al., 2001; Cannon and Leighton, 2004). One simple result of the model is clear: without variable mating behaviors, meta-communities always become dominated by a single species through stochastic population dynamics. Mitigating processes *must* exist that allow rare species to escape extinction. As mentioned above, previous ecological models have identified deterministic, largely density-dependent, factors that help maintain community diversity. Our results indicate that additional evolutionary factors can play a role as well.

Little is known about the stability of population size of species in highly diverse communities through long periods of time, but given millennial dynamics, common species in today’s forests have probably been rare in the past, if not across their entire distribution. Local rarity, where an individual of a species finds itself in a community devoid of conspecifics, must occur in the history of almost all species. Most tree species can be frequently found growing outside their “preferred” habitat. By hindcasting current species distribution models of >300 Dipterocarpaceae species on the geographic and climatic conditions at the last glacial maximum, Raes et al. (2014) found that while most species could persist during the glacial period, their predicted historical abundances were substantially different than current abundances for many species. These millennial dynamics of climate and community change obviously have played a major role in the evolutionary history of these forests. The benefits of maintaining some level of interspecific fertility has probably affected most species in these communities, as no one species truly dominates or remains common over millennial periods of time and across its entire distribution.

Our results potentially have implications for the general understanding of species co-existence and the management of rare species in highly diverse communities. Previous work indicates that the processes of competitive exclusion in diverse communities are inefficient and protracted (Hubbell, 2006). The assumption that reproductive isolation between different species ultimately provides a long-term selective advantage to individuals, the basic premise of the Biological Species concept, has been demonstrated in simple scenarios that assume consistent selection pressures in relation to life history strategy, but this basic assumption has *never* been proven for long-lived species in highly diverse communities dominated by high degrees of uncertainty in the selective environment. We argue that while a unique and complex suite of phenotypic traits may provide an “instantaneous” competitive advantage in a particular ecological community (Davies et al., 1998), the advantage gained by a particular phenotype over long periods of time is unpredictable and frequently, what was advantageous can become disadvantageous. Instead, species identity, as determined by its genealogy, may be more fluid and dynamic through time and space, particularly as the rate of change and spatial heterogeneity in the environment increases in relation to the demographic turn-over in the community, particularly if the suite of phenotypic traits are not tightly linked genetically with traits that also promote assortative mating among phenotypes. Our model applies primarily to species in highly diverse communities, where numerous suites of genomic mutualists are embedded within a much larger and diverse community and each population contributes a relatively small proportion to the entire community. Below a certain level of diversity in the community, these mechanisms likely play a minor role, only affecting the small number of species.

A striking aspect of tropical forest diversity is our limited taxonomic knowledge of many major families (see http://floramalesiana.org/ for a list of families which have never been treated systematically in the Flora Malesiana project, which was initiated over 60 years ago). Many groups have defied strict taxonomic treatment, like the Myrtaceae family, despite the best efforts of dedicated taxonomists. Additionally, diverse groups display a wide range of ecological and evolutionary characteristics. Examples of major tree genera in Southeast Asian forests include: (1) *Litsea*, in the laurel family, with small, frequently unisexual, and primitive flowers producing largely bird-dispersed fruits; (2) *Aglaia* or *Dysoxylum*, both in the Meliaceae family, which produce profuse displays of minute flowers and produce a wide variety of fruit types; (3) *Ficus*, in the Moraceae, with its highly specialized inflorescence and obligate symbiosis with its pollinating wasps; and (4) *Diospyros*, in the Ebenaceae, in which individuals are mostly unisexual (species are dioecious), flowers are large, open, and showy, and most trees exist in the understory. Few traits seem to link these diverse plant groups except substantial potential for hybridization in mixed communities, where a large fraction of the species are rare (Ashton, 1988a), live sympatrically with congeneric species (LaFrankie et al., 1995; Cannon and Leighton, 2004), and mate choice is frequently limited (Ashman et al., 2004; Vamosi et al., 2006). We would suggest that reticulate evolution is a viable explanation for the challenges of species description and identification.

Most detailed pollination studies in these forests also indicate that generalist pollinators are dominant (Momose et al., 1998; Sharma and Shivanna, 2011). Even within genera that show clearly divergent floral morphologies, hybridization still occurs (Zjhra, 2008). Flowering time has been shown to be slightly staggered among sympatric *Shorea* species (Ashton, 1988b), but considerable overlap in floral receptivity exists (Kettle et al., 2011). In less diverse communities, such as temperate forests, successful species truly dominate the community and can gain an advantage through reproductive isolation to prevent the donation of pollen or advantageous alleles to rare species. Examples of persistent inter-specific hybridization in such communities, e.g., oaks, may reflect the influences of other, possibly phylogenetic or ecological factors (Muller, 1952; McCauley et al., 2012), and may play a smaller role in the maintenance of community diversity (Howard et al., 1998; de Casas et al., 2007).

The results of our analysis strongly suggests that variable mating behavior may be an important force in maintaining community level species diversity, primarily by lowering the vulnerability of rare species to stochastic extinction. This behavior also has major implications for the management of rare and endangered species. Given our model, gene flow among genomic mutualists, to both expand population size and to increase genetic variance in the offspring, may be the most effective mechanism for preventing their local extinction and for enhancing their ability to adapt to novel environmental conditions. While little is known about the possible impact of such gene flow, future environmental conditions in the tropics will be largely unprecedented in the Quaternary Period (Corlett, 2012), with global warming pushing climates into the warmest conditions seen since the Eocene. Given global climate and land use change patterns, current habitats are unlikely to persist for even in the near future, making hybrid and novel phenotypes important to the persistence of local endemics and rare endangered species (Guo, 2014).

Ultimately, above and beyond the model presented here, we feel that variable mating behavior may play a major role in tropical diversification as well. To satisfy the standard allopatric or parapatric model of speciation, an elaborate series of biogeographic events must be devised to explain how all of these species would have become subdivided and spatially isolated, or at least parapatric, for evolutionarily significant periods of time. While sympatric speciation seems possible under certain circumstances, the theoretical requirements are stringent (Gavrilets and Vose, 2007; Fitzpatrick et al., 2009) and probably not generally found in highly diverse communities, particularly for long lived organisms with multiple overlapping generations such as rain-forest trees and coral reefs. In these organisms, the demographic integration of ecological and evolutionary dynamics can extend over centuries (Laurance et al., 2004; Petit and Hampe, 2006; Issartel and Coiffard, 2011). For the ecological speciation model to achieve complete reproductive isolation in the tropical setting, the selective forces would have to be quite strong, because the probability of inter-specific gene flow is quite high and the barriers to gene flow would have to evolve rapidly, during the unusual phases when a species is found in isolation. We do not refute that strong selection pressures can cause divergence among sympatric and closely-related taxa (Fine et al., 2013; Misiewicz and Fine, 2014) but rather question whether this process will frequently lead to complete reproductive isolation and whether this fixed endpoint is actually advantageous. A non-zero level of inter-fertility may play a substantial role over evolutionarily significant periods of time.

Our results suggest that inter-specific introgression may play a novel role, by linking two or more lineages through a genomic mutualism periods of community change or fluctuating selection pressures. When conditions are stable and the forces driving ecological speciation are consistent, hybrid offspring would be disadvantageous and reproductive isolation mechanisms would strengthen but when conditions change, even on the scale of hundreds of years, these hybrids can play a critical role in allowing rare or less fit species to capture advantageous alleles through backcrossing and introgression. Additionally, critically rare species, on the verge of local extinction, may play a considerable role in diversification by generating transgressive phenotypes through the greater increased rate of hybrid crosses attempted (Givnish, 2010). Finally, no evolutionary theory can adequately explain the general process of diversification in tropical tree communities, given their evolutionary and ecological setting, where sympatric speciation appears to be the rule, not the exception. Variable mating strategies and genomic mutualisms may provide an evolutionary mechanism for tropical diversification, not only for its maintenance.

### Conflict of Interest Statement

The authors declare that the research was conducted in the absence of any commercial or financial relationships that could be construed as a potential conflict of interest.
